# A pilot survey into the landscape of neuro-oncology care in the community

**DOI:** 10.1093/oncolo/oyaf047

**Published:** 2025-03-31

**Authors:** Christine Lu-Emerson, Sajeel Chowdhary, Rupesh Kotecha, Akanksha Sharma, Yazmin Odia, Brian Vaillant, Charles Redfern, Aaron Mammoser, Kent Shih, Santosh Kesari, Richard Peterson, Bret Friday, W Jeffery Edenfield, Sebastian Koga, James Snyder, Jerry Jaboin, Isaac Melguizo-Gavilanes, Melissa McCabe, Michael Humeniuk, Prakash Ambady, Erin Dunbar

**Affiliations:** Department of Neurology, Maine Medical Partners and Maine Health Cancer Care, South Portland, ME 04106, United States; Department of Neuro-Oncology, Tampa General Hospital Cancer Institute, University of South Florida, Tampa, Florida 33606, United States; Department of Radiation Oncology, Miami Cancer Institute, Baptist Health South Florida, Miami, FL 33176, United States; Pacific Neuroscience Institute and Providence Saint John’s Cancer Institute, Santa Monica, CA 90404, United States; Department of Neuro-oncology, Miami Cancer Institute, Baptist Health South Florida, Miami, FL 33176, United States; Department of Neurology, University of Texas at Austin, Austin, TX 78712, United States; Laurel Amtower Cancer Institute, 3075 Health Center Dr, San Diego, CA 92123, United States; Department of Neuroscience and Oncology, Piedmont Brain Tumor Center, Piedmont Atlanta Hospital, Atlanta, GA 30309, United States; Tennessee Oncology, Plaza II, Nashville, TN 37203, United States; Pacific Neuroscience Institute and Providence Saint John’s Cancer Institute, Santa Monica, CA 90404, United States; Regions Hospital, St. Paul, MN 55101, United States; Essentia Health, Duluth, MN 55805, United States; Prisma Health Cancer Institute, 65 International Dr, Greenville, SC 29615, United States; Koga Neurosurgery, STE C, Covington, LA 70433, United States; Department of Neurosurgery and Neurology, Hermelin Brain Tumor Center, Henry Ford Health, Detroit, MI 48202, United States; University of Oklahoma Health Sciences Center, Oklahoma City, OK 73104, United States; Department of Neuroscience and Neuro-Oncology, Aurora Cancer Care, Milwaukee, WI 53215, United States; Good Samaritan University Hospital, West Islip, NY 11795, United States; Gibbs Cancer Center, Spartanburg Regional Healthcare System, Spartanburg, SC 29303, United States; Providence Brain & Spine Institute, Portland, OR 97225, United States; Department of Neuroscience and Oncology, Piedmont Brain Tumor Center, Piedmont Atlanta Hospital, Atlanta, GA 30309, United States

**Keywords:** community provider, neuro-oncology practitioner, neuro-oncology care, accessibility of clinical trials, multidisciplinary

## Abstract

**Background:**

The complexities of the field of neuro-oncology require multidisciplinary collaboration in order to deliver contemporary comprehensive care. There is increasing awareness that much of neuro-oncology care occurs in the community setting. In 2022, the Society for Neuro-Oncology (SNO) created the Community Neuro-Oncology Committee (CNO) in an inaugural attempt to formally acknowledge community neuro-oncology practitioners.

**Methods:**

A 19 question survey was developed by SNO-CNO to gather initial data on the current landscape of neuro-oncology care in the community. The survey was distributed via the SNO newsletter and email blasts as well as through partnerships with multiple advocacy groups. Results were analyzed and tabulated through *R*^2^.

**Results:**

There were 112 responses from providers in the United States and Canada. Most providers were physicians and represented multiple disciplines including neurology, neuro-oncology, medical oncology, neurosurgery, and radiation oncology. Sixty-four (57%) described themselves as neuro-oncology-focused. Eighty-eight (79%) reported access to neuro-oncology tumor boards. Sixty-eight (73%) stated they had access to molecular tumor boards. Most respondents felt that they were adequately supported to manage neuro-oncology patients. When dividing responses based on a neuro-oncology-focused practice compared to a less neuro-oncology-focused practice, there were significant differences between access to molecular tumors boards (85% vs 63%, *P* = .023) and access to clinical trials (98% vs 82%, *P* = .022).

**Conclusion:**

This qualitative and quantitative hypothesis-generating data is the start of understanding the challenges faced by community neuro-oncology providers. These results will guide future studies and recommendations aimed toward better supporting them and their patients.

**Implications for practice**

This survey is the first known collaborative effort to systematically gather qualitative and descriptive quantitative information about neuro-oncology care provided by predominately community providers. Responses to the survey indicate interest and the importance of this topic. The authors believe that these initial results will inform the current understanding of the unique challenges faced by community neuro-oncology providers and will generate recommendations aimed toward better-supporting providers and their patients. Comparing the results with data from academic settings may identify gaps and opportunities leading to improved care for all patients.

## Introduction

The field of neuro-oncology is incredibly complex, requiring a multidisciplinary approach to deliver appropriate comprehensive care in the modern era. This involves a multidisciplinary team that is generally comprised of neuro-oncology and medical oncology specialists, neurosurgeons specialized in tumor cases, radiation oncologists who are preferentially disease-site subspecialized, neuroradiologists, and neuropathologists with expertise in molecular diagnostics. Support services such as social work, therapy specialties and palliative medicine physicians are often part of the multidisciplinary team. While most people assume that neuro-oncology patients seek care from large tertiary or quaternary academic centers, there is increasing awareness that the majority of neuro-oncology care in the United States occurs in a community setting, especially when considering metastatic brain tumor management. The 2022 Central Brain Tumor Registry of the United States (CBTRUS) data revealed that much of the highest annual age adjusted incidence rates of malignant brain tumors were located in rural states, which correlated with smaller concentrations of dedicated traditional academic brain tumor centers compared to their urban counterparts.^1^ Anecdotally, patients with access to a tertiary or quaternary center do so for a limited time resulting in the majority of patient care, including hospice and palliative services, occurring in the community setting. Understanding the current landscape of the delivery of neuro-oncology care in the community is crucial not only to identify challenges faced by our community partners but to direct understandings toward a call for adequate resources for community neuro-oncology. In 2022, the Society for Neuro-Oncology (SNO) launched an effort to acknowledge community neuro-oncology practitioners by creating the Community Neuro-Oncology (CNO) committee. Inaugural goals of CNO include gathering data to recognize the work of community neuro-oncologists, reviewing the care delivered in this setting, and identifying areas that may benefit from access to disease-specific clinical trials and health equity. The SNO-CNO committee also sought to gather information related to the availability of resources needed to navigate the complex care required by this patient population. This included access to clinical trials, neuro-oncology tumor boards, molecular testing, compassionate use drugs, and exposure to neuro-oncology conferences.

## Methods and materials

**Figure 1.**
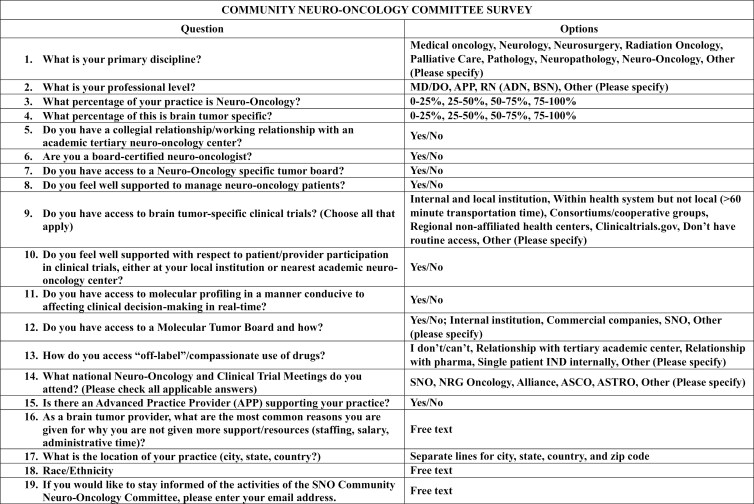
Community neuro-oncology survey.

A search of the literature performed in November 2022 failed to reveal an available instrument. Thus, a de novo survey was created for this study. The survey was initially developed by the two chairs (C. Lu-Emerson and S. Chowdhary) of the SNO-CNO committee. Through iterative revisions and initial reviews by the 23 members of the SNO-CNO committee, 19 question stems were created. Based on the initial intended population (members of the SNO), question choices were evaluated by members of the SNO-CNO subcommittee for question length, adequacy of choices, readability, comprehensibility, and reproducibility. Participants of the survey were also given multiple opportunities to provide additional free text responses to specific questions. For example, participants were asked to share as much as possible regarding resources and support received in caring for their neuro-oncology patients. This 19-item survey was subsequently delivered to members of SNO via the society newsletters and email blasts every 3 months from February 2023 through November 2023 (Figure 1). The survey was accompanied by an introductory letter explaining the purpose of the survey. To assist in identifying providers, we partnered with national and international advocacy groups (ie, American Brain Tumor Association, Brain Tumor Network, National Brain Tumor Society, The Sontag Foundation, The Musella Foundation) who allowed us to distribute the survey to community providers who may not be active in SNO. In August of 2023, the survey was also distributed via the National Cancer Institute (NCI) Community Oncology Research Program (NCORP) division of NRG Oncology in an additional effort to reach oncology members in the community. Other societies and cooperative groups were contacted for permission to distribute the survey but did not respond to inquiries or declined. Because this survey sought providers who self-identified as providing community neuro-oncology care, there were no attempts to verify self-identification or individual responses to questions.

Raw survey data results were tabulated, imported into, and analyzed in *R^2^*. Due to the heterogeneity of answers to the question of “timely access to molecular profiling data,” answers were re-coded into one of 3 categories at the author’s discretion: “yes,” “no,” or ‘limited.’ A comparison of responses was performed using the chi-squared test.

As the survey was approved by SNO for use and dissemination to its members, Institutional Review Board (IRB) approval was granted through the Piedmont Healthcare System IRB in Atlanta Georgia (IRB 2053698-1). Informed consent was waived as this was a voluntary survey distributed to providers involved in neuro-oncology care.

## Results

Over the course of nine months (2/2023-11/2023), 200 respondents from various countries and continents participated in the survey. The majority of practitioners (64%, *n* = 112) were from the United States and Canada, and this initial analysis focused on this population. These respondents were composed of a variety of race/ethnicity groups, including 72% White/Caucasian, 22% Asian, 2% Hispanic/Latino, 0% Black/African American, 1% American Indian/Alaska Native, and 1% Native Hawaiian/Pacific Islander.

The 112 respondents represented a diverse background with 21 (19%) citing neuro-oncology as their primary discipline, 22 (20%) identifying as neurosurgeons, 16 (14%) as neurologists, 20 (18%) as radiation oncologists, and 18 (16%) as medical oncologists (Table 1). The other disciplines (13%) represented included palliative care, pathology, neuropathology, pediatric oncology, and pediatric neuro-oncology. Most respondents (*n* = 94, 84%) represented physicians (MD/DO) with others identifying as advanced practice providers (n = 6, 5%) and nurses (*n* = 12, 11%). A minority (*n* = 34, 30%) of respondents stated that they were board-certified in neuro-oncology.

**Table 1.** Demographics and practice details of provider participants

Characteristics	Neuro-oncology (*n* = 21)^a^	Neurology (*n* = 16)	Radiation Oncology (*n* = 20)	Neurosurgery (*n* = 22)	Medical Oncology (*n* = 18)	Other^b^ (*n* = 15)
**Professional level** **MD** **APP** **RN**	11 (52%) 5 (24%) 5 (24%)	15 (94) 0 (0%) 1 (6%)	19 (95%) 0 (0%) 1 (5%)	19 (86%) 0 (0%) 3 (14%)	18 (100%) 0 (0%) 0 (0%)	12 (80%) 1 (7%) 2 (13%)
**Neuro-oncology-focused** **0–25%** **25–50%** **50–75%** **75–100%**	0 (0%) 2 (10%) 1 (5%) 18 (86%)	3 (19%) 0 (0%) 0 (0%) 13 (81%)	3 (15%) 1 (5%) 5 (25%) 11 (55%)	4 (18%) 4 (18%) 7 (32%) 7 (32%)	2 (11%) 2 (11%) 3 (17%) 11 (61%)	7 (47%) 2 (13%) 2 (13%) 4 (27%)
**Board certified in Neuro-oncology (MD/DO only)** **Yes** **No**	10 (91%) 1 (9%)	14 (93%) 1 (7%)	1 (5%) 18 (95%)	3 (16%) 16 (84%)	4 (22%) 14 (78%)	2 (17%) 10 (83%)
**Access to Neuro-oncology tumor board** **Yes** **No**	18 (86%) 3 (14%)	15 (94%) 1 (6%)	17 (85%) 3 (15%)	15 (68%) 7 (32%)	11 (61%) 7 (39%)	12 (80%) 3 (20%)
**Access to clinical trials** ^c^ **Internal** **Consortium** **Other** **None**	16 (76%) 8 (38%) 12 (57%) 1 (5%)	11 (69%) 8 (50%) 7 (44%) 3 (19%)	14 (70%) 14 (70%) 9 (45%) 0 (0%)	11 (50%) 7 (32%) 7 (32%) 7 (32%)	8 (44%) 6 (33%) 11 (61%) 3 (17%)	8 (53%) 11 (73%) 9 (60%) 1 (7%)
**Access to molecular profiling** **Yes** **Limited** **No**	***N* = 19** 14 (74%) 0 (0%) 5 (26%)	***N* = 15** 12 (80%) 1 (7%) 2 (13%)	***N* = 20** 14 (70%) 0 (0%) 6 (30%)	***N* = 21** 14 (67%) 0 (0%) 7 (33%)	***N* = 18** 16 (89%) 1 (6%) 1 (6%)	***N* = 15** 15 (100%) 0 (0%) 0 (0%)
**Access to molecular tumor board** **Internal** **Commercial** **SNO** **Other** **No**	***N* = 19** 9 (47%) 2 (11%) 1 (5%) 2 (11%) 5 (26%)	***N* = 13** 9 (69%) 2 (15%) 1 (8%) 1 (8%) 0 (0%)	***N* = 16** 5 (31%) 1 (6%) 2 (13%) 0 (0%) 8 (50%)	***N* = 17** 6 (35%) 2 (12%) 2 (12%) 0 (0%) 7 (41%)	***N* = 14** 8 (57%) 3 (21%) 0 (0%) 0 (0%) 3 (21%)	***N* = 14** 8 (57%) 2 (14%) 0 (0%) 2 (14%) 2 (14%)
**Access to off label use/compassionate use of drugs** **Pharmaceutical** **Single IND** **Rel with tertiary center** **Other** **No access**	***N* = 19** 7 (37%) 6 (32%) 1 (5%) 3 (16%) 2 (11%)	***N* = 16** 4 (25%) 2 (13%) 2 (13%) 5 (31%) 3 (19%)	***N* = 20** 1 (5%) 2 (10%) 1 (5%) 0 (0%) 16 (80%)	***N* = 21** 4 (19%) 0 (0%) 2 (10%) 1 (5%) 14 (67%)	***N* = 17** 7 (41%) 3 (18%) 1 (6%) 2 (12%) 4 (24%)	***N* = 14** 3 (21%) 8 (57%) 0 (0%) 3 (21%) 0 (%)
**Attendance of meeting** **SNO** **NRG** **Alliance** **ASCO** **ASTRO** **Other**	***N* = 18** 17 (94%) 2 (11%) 2 (11%) 8 (44%) 0 (0%) 3 (17%)	***N* = 16** 15 (94%) 1 (6%) 3 (19%) 6 (38%) 0 (0%) 1 (6%)	***N* = 18** 6 (33%) 7 (39%) 0 (0%) 1 (6%) 17 (94%) 2 (11%)	***N* = 16** 12 (75%) 1 (6%) 0 (0%) 0 (0%) 1 (6%) 5 (31%)	***N* = 17** 9 (53%) 0 (0%) 2 (12%) 12 (71%) 1 (6%) 1 (6%)	***N* = 13** 11 (85%) 0 (0%) 0 (0%) 1 (8%) 0 (0%) 6 (46%)
**Access to APP** **Yes** **No**	***N* = 21** 17 (81%) 4 (19%)	***N* = 16** 10 (63%) 6 (38%)	***N* = 20** 6 (30%) 14 (70%)	***N* = 22** 19 (86%) 3 (14%)	***N* = 18** 14 (78%) 4 (22%)	***N* = 15** 9 (64%) 6 (43%)
**Region** **Northeast** **Midwest** **South** **West** **Puerto Rico** **Canada**	***N* = 21** 5 (24%) 4 (19%) 5 (24%) 7 (33%) 0 (0%) 0 (0%)	***N* = 16** 4 (25%) 2 (13%) 2 (13%) 8 (50%) 0 (0%) 0 (0%)	***N* = 20** 8 (40%) 2 (10%) 3 (15%) 4 (20%) 0 (0%) 3 (15%)	***N* = 22** 5 (23%) 4 (18%) 8 (36%) 2 (9%) 2 (9%) 0 (0%)	***N* = 18** 3 (17%) 3 (17%) 6 (33%) 5 (28%) 0 (0%) 1 (6%)	***N* = 15** 5 (33%) 2 (13%) 2 (13%) 4 (27%) 0 (0%) 2 (13%)

^a^One respondent was board certified in both neuro-oncology and medical oncology.

^b^Includes Palliative Care, Pathology, Neuropathology, pediatric oncology, general practitioner, pediatrics, pediatric neuro-oncology.

^c^Respondent may have multiple answers.

Ninety-eight (88%) attested to attending at least one national meeting. Of these, 70 (71%) reported attending the annual SNO meeting; 28 (29%) reported attending the annual ASCO meeting; 19 (19%) reported attending the annual ASTRO meeting; 11 (11%) reported attending an NRG meeting; and 7 (7%) reported attending at least one Alliance meeting. Fifty (51%) respondents cited attending more than one national meeting per year.

Of 112 responses, 64 (57%) stated that 75%-100% of their practice was neuro-oncology focused. On the other end of the spectrum, 19 (17%) stated that neuro-oncology was limited to 0%-25% of their practice. It is important to note that the survey did not provide a strict definition for a neuro-oncology-focused practice and left this to the interpretation of the respondent, who self-identified as a community neuro-oncology provider. Thirty-nine people responded to the question regarding brain tumor-specific practice. Thirty-two practitioners (82%) reported that 75%100% of their practice was brain tumor-specific. Despite the cover letter stating the goal was to identify community providers, respondents to the surveys comprised a mix of self-identified academic centers and community practice, with 5 (5%) identifying as working at tertiary centers. Of the 112 people who answered the question regarding access to a neuro-oncology-specific tumor board, the majority (*n* = 88, 79%) stated that they did have access to these tumor boards. However, the survey did not discern if tumor board access was internal versus via a commercial entity or virtual tumor board.

A question asked if respondents felt adequately supported to manage neuro-oncology patients. This question was intentionally designed to be self-interpreted by the respondent regarding their definition of current adequate support (eg, staffing, lab or clinic space, protected administrative time). Of 111 respondents, 88 (79%) reported feeling adequately supported. There were 112 responses to the question about access to brain tumor-specific clinical trials. 68 (61%) stated that they had access to internal and local institution trials; 45 (66%) of these respondents stated that they also had access to trials via consortiums and cooperative groups. Eight people (7%) had access to trials only via consortium and cooperative groups. Fifteen (13%) responded stated that they did not have routine clinical trial access. A follow-up question gauging support for participation in trials received 109 responses. Most affirmed support but some qualified the question with comments about the limited availability of trials. When asked the question if there is an APP supporting their practice, 37 (33%) of 112 respondents reported a lack of APP support. Radiation oncology was the most common discipline to cite a lack of APP support (*n* = 14 out of 20 responses, 70%). The most common reasons cited for lack of support were fiscal, staffing, and insurance reasons.

With the updated diagnostic WHO 2021 criteria, recognition of the need for molecular diagnostics has increased.^3^ Access to molecular profiling in real time was reported by 87 (81%) of the 108 respondents, but 21% stated that they had no or limited access to timely molecular profiling. Of note, the question was designed for responders to self-interpret, based on their personal definition of adequate “access”; the question did not define “real time” nor did it specify what entailed “molecular profiling.” Access to a molecular tumor board was reported by 68 out of 93 respondents (73%) in a follow up question. Of those having access, 45 (66%) reported availability of molecular tumor board through their internal institution, while 23 (33%) stated they had external resources. Twenty-five (27%) stated that they did not have access to molecular tumor boards.

We reviewed responses divided between providers who self-identified as neuro-oncology focused (NOF), defined as a practice consisting of 75% or more dedicated to neuro-oncology, compared to respondents who self-identified their practice as comprised of less than 75% neuro-oncology (NOL) (Table 2). Regarding access to timely molecular profiling, NOF and NOL both felt that they had timely access to some sort of profiling (83% vs. 82%, *P* = .694). There was a significant difference between NOF and NOL in regard to access to molecular tumor board (85% vs 63%, *P* = .023) and access to trials (98% vs 82%, *P* = .022). Finally, there was a statistically significant difference between NOF and NOL regarding support from an advanced practice practitioner (79% vs 58%, *P* = .024). Analyses were not conducted to understand other factors as potential covariates.

**Table 2.** Access

	NOF^a^ (%)	NOL^b^ (%)	*P* ^c^
**Access to timely molecular profile** • Yes • No • Limited	*N* = 46 36 (78) 8 (17) 2 (4)	*N* = 62 50 (81) 11 (17) 1(2)	.694
**Access to clinical trial** • Yes - Internal/local access • No	*N* = 47 46 (98) 40 (87) 1 (2)	*N* = 65 53 (82) 33 (62) 11 (17)	.023
**Access to molecular tumor board** •Yes - Commercial - Internal - SNO •No	*N* = 39 33 (85) 4 (12) 23 (70) 2 (6) 6 (15)	*N* = 54 34 (63) 8(24) 18 (56) 4 (12) 20(37)	.022

^a^NOF: neuro-oncology (focused) practice comprised of ≥75% of overall practice.

^b^NOL: neuro-oncology practice comprised of <75% of overall practice.

^c^χ^2^.

## Discussion

This was an exploratory survey study designed to gather hypothesis-generating qualitative and descriptive quantitative data through the formal solicitation of those who identified themselves as community neuro-oncology providers. In this pilot survey, there was no attempt to use data from other work force surveys as it was not felt to be representative or additive. In order to capture the individual perspectives of responders, the phrasing of some questions and the options for answering, were intentionally broad to allow for self-interpretation. For example, the phase “adequate access to clinical trials” did not have a strict definition. Given the intended self-identification and self-reporting nature of this pilot study, there was no attempt to verify responses.

We are humbled by the number of responders to our survey. Although we feel this represents an important foundational step in understanding the landscape of the community neuro-oncology practice, we acknowledge several limitations that will be better addressed in future initiatives. For instance, 15 of the 112 respondents (13%) skipped the question regarding race and ethnicity. While 0 of the 97 respondents to the question answered Black/African American, this could be a product of self-reflection from a convenience sample. Most respondents were reached via distribution of the survey to SNO members, raising concern SNO members may not fully capture the target audience of true community providers caring for neuro-oncology patients. While most respondents self-identified as in community practice, a surprising number stated that most (75%-100%) of their practice was neuro-oncology focused. The 5 respondents who associated themselves with an academic or tertiary center were included in the NOF group. There are ongoing efforts to expand the survey to the appropriate audience. Strengthening partnerships with advocacy groups may provide alternative ways of revealing the demographics around community neuro-oncology care. Access to prescription patterns and pharmacologic dispensation of temozolomide may provide a more granular way of identifying community neuro-oncology providers, thereby broadening the audience for the survey. The majority of providers identifying as neuro-oncologists or neurologists attested to being board-certified in neuro-oncology. This was not seen in other disciplines including the medical oncologists. This may reflect that board certification is less of a professional priority for these disciplines despite the fact that these subspecialists are eligible for certification. Another important and unexpected finding from the survey was that most respondents reported feeling adequately supported to manage their current neuro-oncology patients. Yet, there was a high number of comments reporting a desire for increased personnel and infrastructure. This discrepancy may reflect both wide variations in the personal/professional characteristics of providers, as well as wide variations in their operational practices. The SNO-CNO hopes to better characterize the factors behind this survey’s discrepant and unexpected findings in future initiatives. One potential mechanism to expand on this issue includes a follow up survey with direct questions about personnel and infrastructure support. Collaboration with other SNO committees such as the External Relations Committee may deepen our understanding of how other organizations, including sister membership societies, and disciplines view neuro-oncology care in the community.

Most respondents indicated that they had adequate access to clinical trials and the ability to conduct clinical trials. This may represent the increased awareness of the importance of trials for neuro-oncology patients due to the limitations of standard treatment. Conversely, this may represent selection bias with the targeted audience not necessarily representative of the true providers in the community caring for neuro-oncology patients. Of note, this survey did not define either the location of clinical trials (institution, local, national, international, via cooperative networks/consortiums or via industry) nor did it define the type of clinical trial (such as brain specific trials, disease agnostic studies, or supportive and palliative care trials). Many commented on the paucity of trial availability in their region. This may reflect higher concentrations of trials at large neuro-oncology institutions typically located in major cities, indicating the need for wider collaborations to enhance inclusivity of the patient population represented in trials and to equalize neuro-oncology care.^4^ One such mechanism may be to further leverage cooperative networks. In 2010, the NCI created the NCORP system, a community based national network designed to provide access to clinical trials, linking academic medical or cancer centers with community sites to reduce systemic inefficiencies.^5^ Another mechanism may involve state-level initiatives such as the Minnesota Cancer Clinical Trials Network to foster clinical trial participation in rural communities.^6^

Interestingly, our survey did not reveal perceived lack of access to molecular diagnostics, assumed to occur in the community. With the updated WHO criteria, molecular diagnostics have played an increasingly important role in the diagnosis of and therapy decision for central nervous system (CNS) tumors. Most respondents endorsed adequate access to molecular profiling in real time including access to molecular tumor boards to allow appropriate interpretation. However, 27% of respondents stated no access to molecular tumor boards with some seemingly unaware of such a resource despite the increased availability of information and education through societies like SNO, the National Institute of Health (NIH) or by commercial platforms. This may be attributed to the fact that the majority of respondents to the survey are affiliated with SNO and may be more neuro-oncology focused than the typical neuro-oncology provider in the community. However, the higher than expected respondents attesting to access to molecular profiling and molecular tumor board could simply reflect the current landscape of oncology care which has increasingly relied on identification of molecular markers to allow for more comprehensive diagnostic classification and to guide therapy. Future investigation into this topic may include a follow up survey to gauge the respondent’s definition of “real time access” to testing. A webinar on molecular profiling and classification may be developed to provide education and to engage with a live audience as to their experience with molecular diagnostics.

## Conclusion

This survey is the first known collaborative effort to systematically gather qualitative and descriptive quantitative information about neuro-oncology care by predominately community providers. The current number of responses indicate interest and the importance of this topic. The SNO-CNO authors believe that these initial results will inform our understanding of the unique challenges faced by community neuro-oncology providers and will generate recommendations aimed toward better supporting providers and their patients. By comparing our results with the much-larger data regarding neuro-oncology care in the academic setting, we hope to reveal gaps and opportunities leading to improved care for all patients. While clinical trials are often cited as a key health inequity between those in the community setting versus a more urban academic setting, access to subspecialized multidisciplinary care through neuro-oncology tumor boards may also be limited in some community care settings. Given that multidisciplinary care, including tumor boards, has been shown to improve neuro-oncology outcomes, the expansion of telehealth tumor boards for community neuro-oncology patients may be an increasingly important way to help bridge the care gap.^7-9^

In the next phase of this work, we look forward to analyzing future responses and incorporating them into a more refined list of future initiatives. Based on connections made through this survey and through complimentary SNO initiatives, we hope to identify more community neuro-oncology providers and lay the groundwork for establishing a multidisciplinary network that supports patients, providers, healthcare colleagues, and care advancements.

## Data Availability

The data underlying this article will be shared on reasonable request to the corresponding author.
